# The progress of Chinese burn medicine from the Third Military Medical University—in memory of its pioneer, Professor Li Ao

**DOI:** 10.1186/s41038-017-0082-z

**Published:** 2017-05-30

**Authors:** Haisheng Li, Junyi Zhou, Yizhi Peng, Jiaping Zhang, Xi Peng, Qizhi Luo, Zhiqiang Yuan, Hong Yan, Daizhi Peng, Weifeng He, Fengjun Wang, Guangping Liang, Yuesheng Huang, Jun Wu, Gaoxing Luo

**Affiliations:** Institute of Burn Research, State Key Laboratory of Trauma, Burn and Combined Injury, Southwest Hospital, Third Military Medical University, Gaotanyan Street no.29, Shapingba District, Chongqing, 400038 China

**Keywords:** Burn injury, Chinese burn medicine, Li Ao, Third Military Medical University, Inhalation injury

## Abstract

Professor Li Ao was one of the founders of Chinese burn medicine and one of the most renowned doctors and researchers of burns in China. He established one of the Chinese earliest special departments for burns at Third Military Medical University (TMMU) in 1958. To memorialize Professor Li Ao on his 100th birthday in 2017 and introduce our extensive experience, it is our honor to briefly review the development and achievement of the Chinese burn medicine from TMMU. The epidemiology and outcomes of admitted burn patients since 1958 were reviewed. Furthermore, main achievements of basic and clinical research for the past roughly 60 years were presented. These achievements mainly included the Chinese Rule of Nine, fluid resuscitation protocol, experience in inhalation injury, wound treatment strategies, prevention and treatment of burn infections, nutrition therapy, organ support therapies, and rehabilitation. The progress shaped and enriched modern Chinese burn medicine and promoted the development of world burn medicine.

## Background

**Fig. 1:**
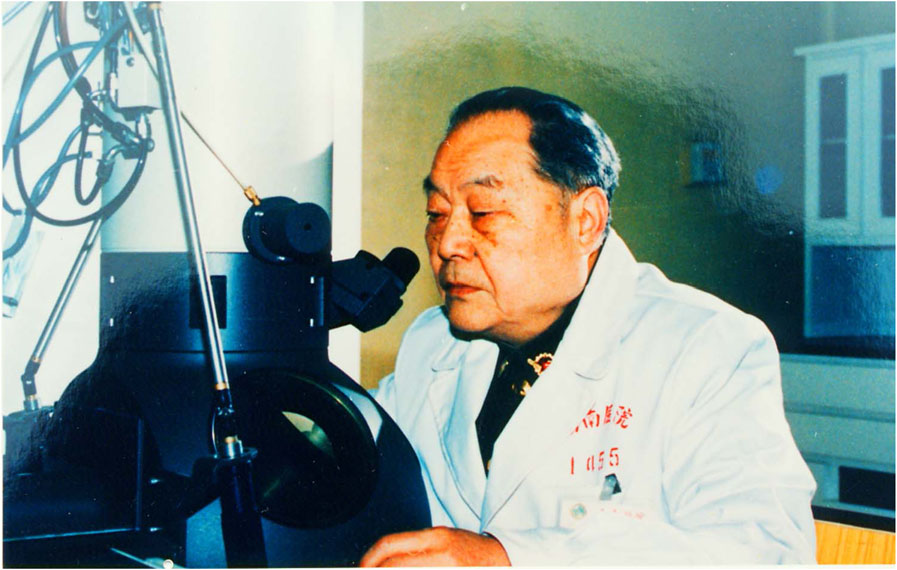
Professor Li Ao at the Institute of Burn Research in 1996

The year of 2017 is the one hundredth anniversary of the birth of Professor Li Ao, who was one of the founders of Chinese burn medicine and one of the most renowned doctors and researchers of burns in China (Fig. 1). In 1958, China entered the period of the Great Leap Forward, which mainly included the massive and unstandardized production of steels. During this process, a vast majority of burn injuries occurred. As a general surgeon, Professor Li Ao realized that burn injuries would become a severe problem in the country. With the help of the Southwest Hospital affiliated with the Third Military Medical University (TMMU), he and his colleagues established the earliest special department for burns, which was the precursor to the Institute of Burn Research (IBR). Although it started with 3 doctors and 6 beds, it now has 30 doctors and 150 inpatient beds (including 18 ICU beds). More than 200 inpatients were admitted at the peak time, and approximately 1800 burn inpatients from the southwest of China and other distant regions are hospitalized at the IBR every year. Since its foundation, over 27,000 burned patients and over 3000 patients undergoing reconstruction and rehabilitation have been admitted to the IBR. In fact, the IBR has become one of the biggest burn centers not only in China but also in the world.

Prompted by Professor Li Ao, the IBR has been continuously exploring the pathophysiological characteristics and the treatment strategies of burn injury for the past 60 years. These studies have covered basic mechanism to clinical treatment, such as burn hypoxia/ischemia injury, fluid resuscitation, inhalation injury, wound healing, burn infection, burn metabolism and nutrition, and hypertrophic scarring. This research shaped and enriched modern Chinese burn medicine and has contributed to burn medicine around the world. The progress of the burn medicine at TMMU and the development of IBR are also attributed to many experts and doctors, such as Professor Xiao Guangxia, Professor Yang Zongcheng, Professor Wang Shiliang, Professor Zhang Yaping, Professor Hu Jia’nian, Professor Ai Shenhai, Professor Kang Shaoyu, and Professor Shi Jixiang.

Because of the achievements in burn medicine, Prof. Li Ao won the Evans Prize from the American Burn Association and was elected to the Chinese Academy of Engineering in 1994. However, he passed away in 1999. To memorialize Professor Li Ao, it is our honor to review the development and achievement of the IBR in burn medicine on the occasion of his 100th birthday.

## Review

### Epidemiology and outcomes

All the literatures related to burn epidemiology in the IBR were reviewed, and the data before 2002 were extracted from five articles [1–5]. The unpublished data were summarized according to the medical records with ethical approval of hospital ethical committee. In total, over 27,000 burned patients and over 3000 reconstruction and rehabilitation patients have been admitted to the IBR. The new admission rate was less than 120 cases a year before 1980, and since then, the number has increased rapidly. The number of inpatients reached roughly 1800 cases in 2016.

#### Burn area

**Fig. 2:**
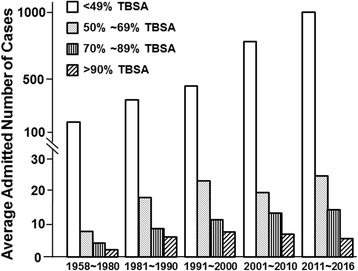
Average annual cases of admitted burn patients with different burn areas

Although the number of burn inpatients has sustained growth, the pattern of cases has been changing. From 1958 to 1990, the cases of both minor (defined as less than 50% total body surface area (TBSA)) and major (defined as over 50% TBSA) burn injuries sustained continuous growth. Since the 1990s, although the number of admitted cases of minor burns has increased rapidly, the cases of major burns remained at an approximately constant level, and the average number of admitted patients with severe burns (over 90% TBSA) even began to decrease (Fig. 2). This is similar to the result of a previous study in the east region of China [6]. Because most cases of major burn injuries occur in the workplace, this trend might reflect the improvement of industrial injury protection in China over past decades.

#### Etiology

**Fig. 3:**
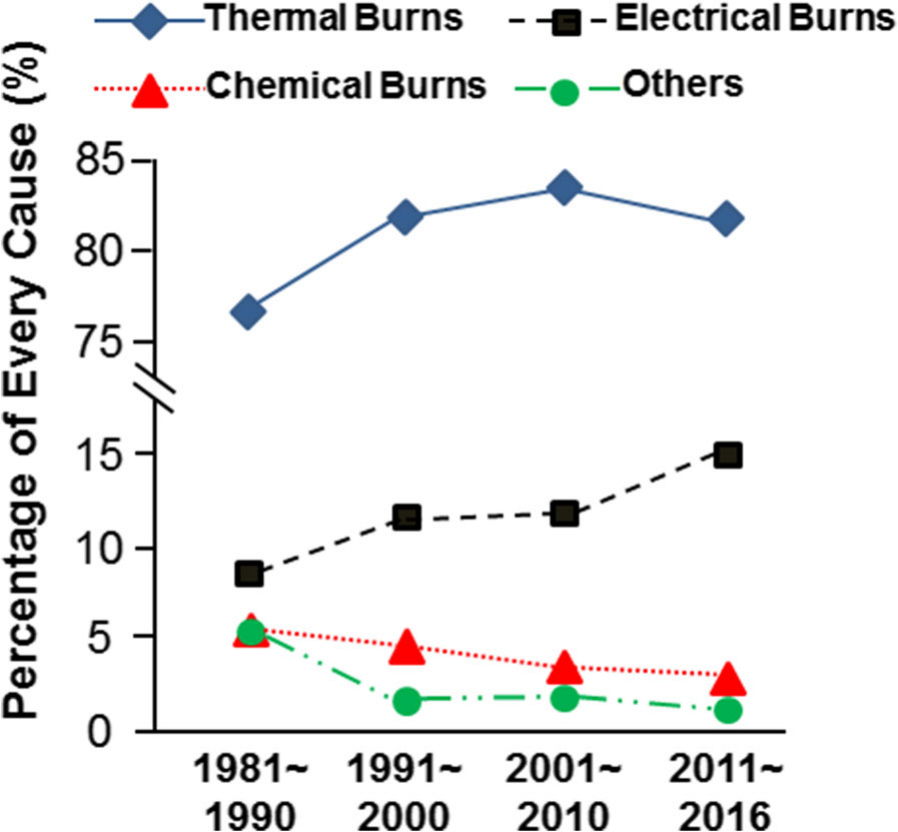
Burn etiology characteristics

The main causes of burn injury have also changed over the past 40 years (Fig. 3). The thermal injury is consistently the leading cause of burns, comprising approximately 80% of all burns. The proportion of burns associated with electrical injury significantly increased from 8.52% in the 1980s to 15.06% in the 2010s (*p* < 0.001). For chemical injury, although the absolute number of cases every year was approximately equal from 1980 to 2010, a reduction in the relative percentage was observed in these four decades. The increased morbidity of electrical injury was a common trend in China and might be associated with the expansion of the power grid in this country and a defect of safety precautions [6, 7]. Given the poorer prognosis and high amputation rate associated with electrical injury, more attention should be given on the prevention of electrical burns [8].

#### Cure rates

**Fig. 4:**
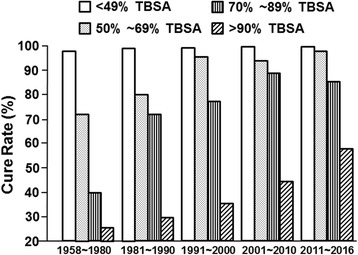
The distribution of cure rates by burn areas

With the fundamental understanding of the pathophysiological changes after burns and the establishment of a treatment system, the therapeutic effect continuously improved, especially since the 1980s. The total cure rate was 92.48% before 1980 and increased significantly to 96.64% in the 1980s (*p* < 0.001). Then, it increased gradually to 99.20% in the 2010s. For minor (<50% TBSA) burn patients, the cure rate maintained a high level at over 90% and increased slightly in these decades. For major (≥50% TBSA) burn injuries, the cure rate rose significantly (Fig. 4). The above results indicate that great improvements in treatment outcomes have been achieved in the IBR.

#### Mortalities

**Fig. 5:**
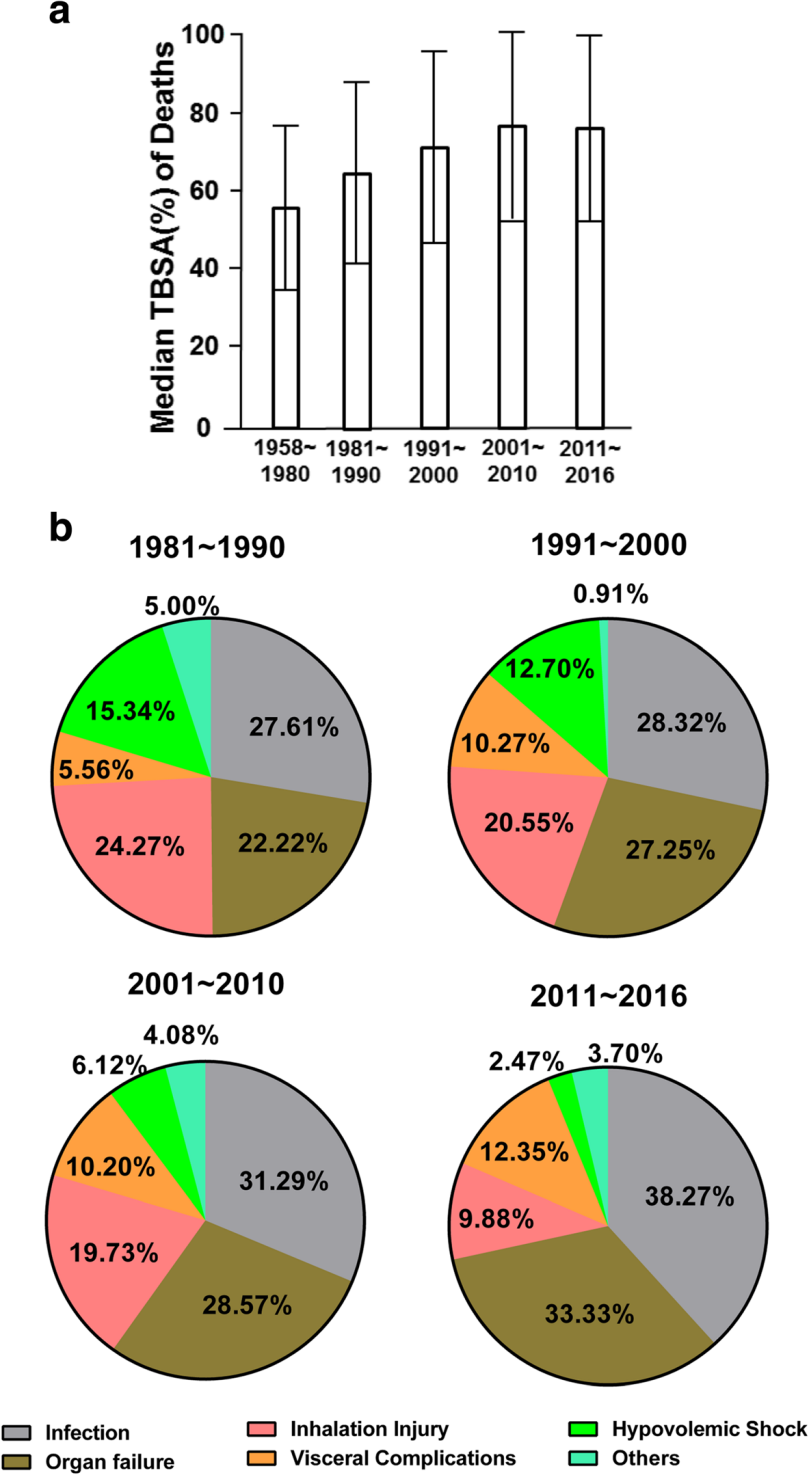
Analysis of burn deaths. **a** The distribution of median TBSA of burn deaths. **b** The causes of burn deaths

With the improvement of burn treatment, the mortality of burn victims has continuously declined in the last 60 years. From 1958 to 1980, the median burn area of patients dying from burns was 58.4% TBSA, and it rose gradually in the 1980s and 1990s, until it reached a plateau of approximately 77% TBSA in the 2000s (Fig. 5a). This also reflects the improvement of the burn treatment.

The cause of death also changed greatly across the decades (Fig. 5b). Systemic infection has been the leading cause of death of burn patients over the past 40 years. The proportion of deaths from infection even increased from 27.61% in the 1980s to 38.27% in the 2010s. Internal organ complication was the second main cause of burn deaths, and the percentage of deaths caused by organ failure increased significantly from 22.22% in the 1980s to 33.33% in the 2010s. However, the proportion of deaths, which are led by inhalation injury and hypovolemic shock, were once the main causes of death in the early stage of burn injury and have continued to decline in the meantime.

### Main achievements of basic and clinical research

Compared with other domestic or foreign burn centers, the IBR appears to have better outcomes of burns, especially for major burn patients. One of the explanations is our extensive experience, which is based on the large number of cases admitted. However, the most important reason might be that the IBR has been focusing on carrying out clinical and basic research and has translated the basic findings into clinical practice. Some of the main achievements obtained from the IBR are as follows.

#### Creating the Chinese Rule of Nine to estimate the burn area

**Fig. 6:**
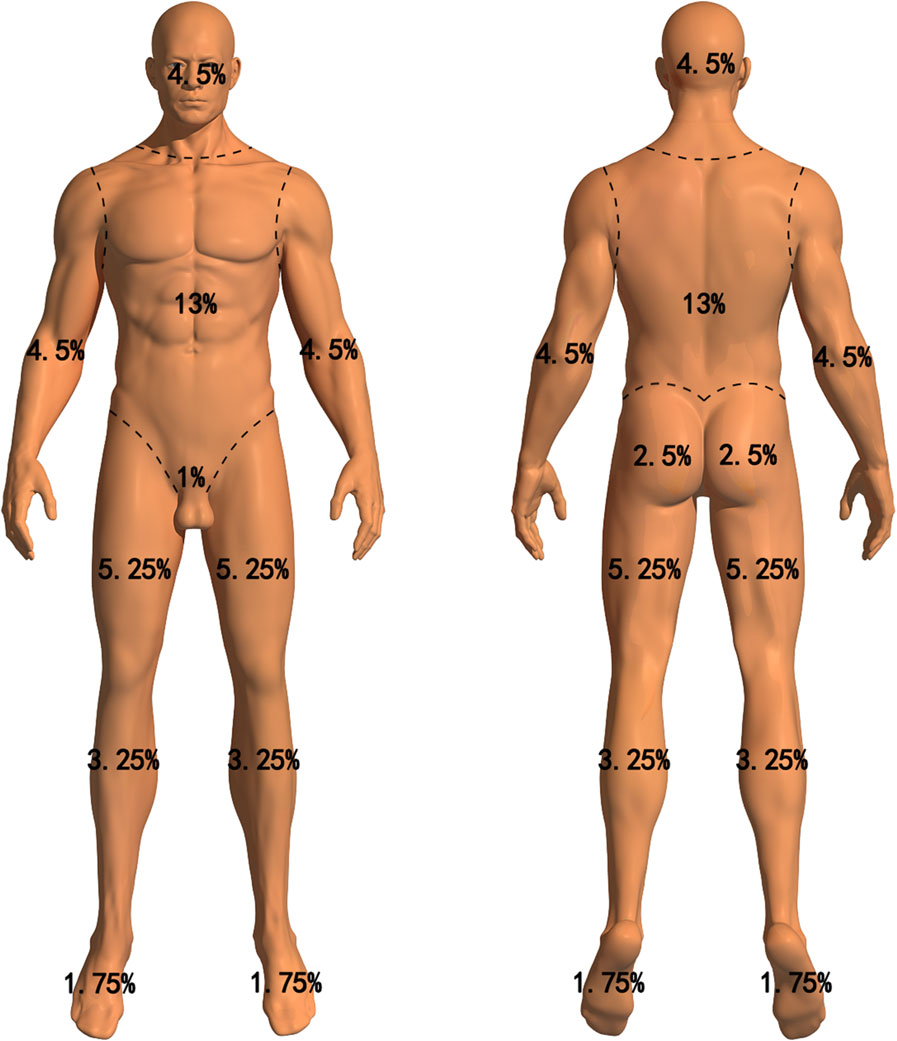
The schematic diagram of the Chinese Rule of Nine

The burn area is one of the major factors when judging burn severity. Based on the measure of body area from 450 healthy Chinese adults, the IBR and colleagues from the histology department of the TMMU developed the Chinese Rule of Nine in the early 1960s [9]. The details of the Chinese Rule of Nine for adults are as follows (Fig. 6): one nine in the head/neck: scalp 3%, face 3%, and neck 3%; two nines in the upper limbs: hands 5%, forearms 6%, upper arms 7%; three nines in the trunk: front 13%, back 13%, perineum 1%; and five nines in the lower limbs: buttocks (male 5%, female 6%), thighs 21%, shins 13%, feet (male 7%, female 6%). Because children have higher area of the head and lower area of the lower limbs than adults, the area of the head/neck equals 9% plus (12–age) % and the area of the lower limbs equals 46% minus (12–age) %. The major difference between the Chinese Rule of Nine and the Wallace rule lies in the assigned area of the trunk and lower limbs. The Chinese Rule of Nine considers a larger area of the trunk and a smaller area of the lower limbs than does the Wallace rule [10, 11]. Because the Chinese Rule of Nine is more suitable for Chinese than the Wallace rule, the Chinese burn committee implemented this rule in 1970. Presently, almost all of the burn centers in China use this Chinese Rule of Nine to estimate the burn area.

#### Forming the Chinese protocol of fluid resuscitation for major burns

The massive fluid leakage from circulation to interstitial space and wound, which is induced by the increased vascular permeability, is the pathophysiological foundation for a decreased circulating volume that may result in hypovolemic shock. Therefore, intravenous fluid resuscitation is often required in the first 48 h following burns, especially in cases of severe burns. Different burn centers have different methods of fluid resuscitation during the early phase after the severe burn injury. Although multiple formulas, such as the Parkland, Evans, and Brook formulas, have been adopted worldwide, there is no international consensus, and the fluid resuscitation protocol varies across regions and burn centers. Based on the experiences of burn fluid resuscitation in 147 adult major burn patients, the IBR developed the TMMU formula in 1962 [12]. Our recent study showed that the TMMU protocol was more suitable for Chinese patients. With the help of primary medical organizations in China, the TMMU formula has been widely used for the fluid resuscitation of severe burns during the early phase.

The TMMU fluid resuscitation protocol contains the following steps [13]. (1) During the first 24 h after injury, 1 ml of crystalloid and 0.5 ml of colloid per 1% TBSA burn area per kilogram body weight and 2000 ml water are given to adult burn patients. For pediatric burn patients, 1 ml of crystalloid and 1 ml of colloid (younger than 2 years) or 0.88 ml of each (older than 2 years) per 1% TBSA burn area per kilogram body weight are given. Furthermore, the water volume is calculated based on the body weight (100 ml per kilogram for the first 10 kg body weight, 50 ml per kilogram for the second 10 kg body weight, 20 ml per kilogram for the rest). For the administering speed, this protocol recommends that one half of the total fluid should be administered within the first 8 h and that the other half be administered in the following 16 h. The common types of colloid include blood, plasma, albumin, and dextran. The recommended crystalloid is lactated Ringer’s solution, and water is 5% glucose solution. (2) During the second 24 h after injury, the TMMU protocol recommends that half of the crystalloid and colloid volume and the same volume of water that were used in the first 24 h be homogeneously given. (3) If the fluid installation was completed in advance, the crystalloid and colloid (ratio of 2:1), not water, should continue to be administered. (4) Doctors should keep in mind that any fluid formula is only the estimation of required fluid volume; thus, the fluid resuscitation should be adjusted based on the situation of the patient, such as age, gender, burn depth, complication, resuscitation starting time, patient’s response, and basic condition. In particular, the speed of fluid resuscitation should be changed according to the comprehensive evaluation, and monitoring of the patient, such as the urine output, mental status, heart rate, blood pressure, and tissue perfusion, should be performed. Furthermore, we also developed a formula for delayed rapid fluid resuscitation for burn patients [14].

**Fig. 7:**
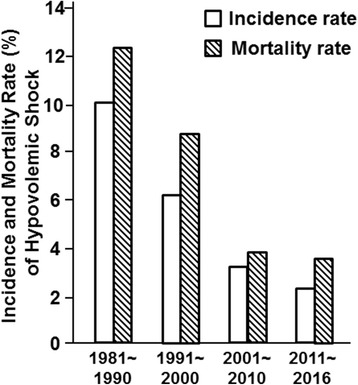
Incidence and mortality of hypovolemic shock

Under the guidance of the TMMU protocol, the incidence and mortality of hypovolemic shock has continued to decrease over the past 40 years. In the 1980s, the overall incidence of hypovolemic shock was 10.14%. In contrast, in the 2010s, the incidence of hypovolemic shock was only 2.26%. The mortality rate of patients with hypovolemic shock dropped from 12.50% during the 1980s to 3.70% during the 2010s (*p* < 0.001) (Fig. 7).

#### Framing the strategies of diagnosis and treatment of inhalation injury

**Fig. 8:**
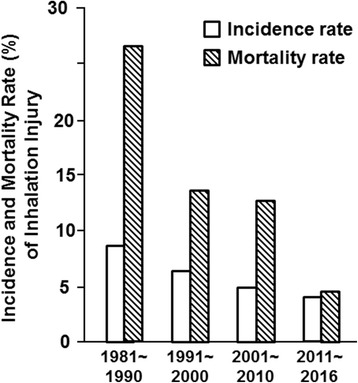
Incidence and mortality of inhalation injury

Our center began experimental research on the mechanism of inhalation injury in the early 1980s [15–17]. Our experimental research and clinical experience promoted the understanding and care of inhalation injury. It was found that the increased pulmonary microvascular permeability, induced by various humoral factors (i.e., lysosomal enzyme), was responsible for the pulmonary edema [18, 19]. Both the incidence and the mortality of inhalation injury have decreased gradually over the past 40 years (Fig. 8). In the 1980s, the overall incidence of inhalation injury was 8.95%, but in the 2010s, the incidence of inhalation injury was only 3.73%. The mortality rate of patients with inhalation injury dropped from 25.40% during the 1980s to 4.47% during the 2010s (*p* < 0.001).

Active care, including early diagnosis, early airway control, and mechanical ventilation, has contributed to the improved clinical treatment effect [20]. The increased application of fiber-optic bronchoscopy aided the accurate diagnosis and severity grading of inhalation injury. In our center, most patients who had a history of exposure to steam, smoke, or chemicals in a closed space and the singeing of hairs in the nose vestibule would receive fiber-optic bronchoscopy. Furthermore, early tracheotomy was performed not only to benefit mechanical ventilation but also to prevent upper airway obstruction; therefore, the rate of tracheostomy was relatively higher. The indication for mechanical ventilation was also not strict. It is always complicated with the dysfunction of microcirculation during the early phase after a severe burn injury. To ensure adequate support of oxygen to cells and tissues, the level of PaO_2_ should be maintained over 100 to 120 mmHg with the application of mechanical ventilation. In addition, other active treatment strategies, such as no restriction of fluid volume in shock resuscitation, inhaling pure oxygen in the first 2 h, early and repeated bronchoalveolar lavage, and intratracheal administration of alveolar surfactant, are encouraged in our center.

#### Forming the Chinese methods to heal burn wounds

##### Early extensive escharectomy

The hypoxia and ischemia induced by burn stress or endotoxemia shock could stimulate the secretion of various cytokines and eventually lead to systemic inflammatory response syndrome (SIRS). SIRS can cause multiple organ dysfunction syndrome (MODS) and even multiple organ failure (MOF), which are two major risk factors for deaths due to burns. Because the necrotized tissues, the edematous fluid, and the infected foci in the burned wounds are the main continuous sources of stimuli, we held the view that escharectomy should be performed as early and thoroughly as possible to contain SIRS. Our research showed that early extensive escharectomy could significantly improve the survival rate, reduce the incidence of MODS and MOF, and decrease the level of various inflammatory cytokines [21]. In our experience, the whole eschar of full-thickness burns and adjacent deep partial-thickness burns were excised at one operation as soon as the hemodynamic parameters and the patient’s general condition were stable. The excised wound beds were covered with skin autografts and a heterogenetic skin graft. It is worth noting that a cooperative expert team (including experienced burn doctors, nurses, and anesthesiologists) and enough grafting skin and blood preparation are the prerequisites for early extensive escharectomy [22, 23].

##### Intermingling auto-allografts and microskin grafts

It is still a big challenge to heal the extensive burn wounds after an escharectomy due to the shortage of autoskin donor sites. There are many methods to cover the extensive skin wounds, such as small piece skin graft, meshed graft, MEEK graft, microskin graft, intermingling auto-allografts, and large sheet allograft. The intermingling of auto-allografts indicates the intermingled transplantation of large sheet alloskin with little pieces of autoskin. First, large sheet alloskin is dispersed by square U holes with an area of 0.5 cm × 0.5 cm and a gap of 1 cm. The holed alloskin is covered on the wound bed. We also invented a machine that could prepare the U holes on the large sheet alloskin. Second, 3 to 5 days later, same-sized split autoskin are embedded into the hole of alloskin and covered with the U alloskin flap of the holes. This method requires large amounts of alloskin.

Micro-autoskin graft is another main method to treat the extensive burn wounds. The microskin graft technique was first reported in 1986 [24] and could economize the autograft. At our institute, microskin grafting was performed regularly in patients who had limited autografts by using the reported methods with some modifications. We found that a microskin graft with an area expansion ratio of 10:1 achieved the best wound healing effect [25, 26]; thus, the microskin graft could partly solve the problem of covering extensive burned wounds with small areas of autografts [27, 28].

Suitable coverage is necessary to supply the best microenvironment for the survival and expansion of the skin autograft on wounds. During the operation of intermingling auto-allografts or the microskin graft, the large sheet alloskin is always necessary. IBR invented a method to prepare the kind of large sheet alloskin in 1962. The large sheet alloskin was obtained by shelling the subcutaneous tissue and partial dermis with a drum dermatome from the cadaver skin continuously without cutting it off. It was found that the alloskin is the best temporary coverage for extensive burn wounds and autografts. However, we must find some other suitable substitute materials due to the shortage of cadaver alloskin.

##### Transgenic porcine skin

Other than autograft skin and cadaver skin, heterogenetic porcine skin graft is the best choice of wound coverage. However, the survival time and effect of porcine skin graft are limited due to the immune rejection. In contrast, the conventional immunosuppressive agents are not suitable for burn patients. CTLA4 Ig could competitively bind with the B7-CD28 and block the B7/CD28-CTLA4 co-stimulation signals, which play a key role in acute cellular rejection after porcine skin transplantation. Therefore, we transferred the CTLA4Ig gene into porcine skin in vitro with the adenovirus vector before transplantation. It was found that CTLA4 Ig could be massively expressed locally in the porcine skin, which prolonged the survival by decreasing the immune rejection and improved its treatment effect [29]. We confirmed its safety by a multi-center, randomized parallel control clinical trial. The gene-transferred porcine skin graft has been approved by the Chinese State Food and Drug Administration and commercialized, and this product is widely used in wound coverage of severe burn patients. Moreover, from the clinical trial, it was found that the average survival time after the gene-transferred porcine skin was prolonged to 19.37 days from 13.63 days of non-transferred control porcine skin. To have a longer survival time, we successfully cultivated a transgenic pig which could express CTLA4Ig specifically only in the skin, and the transgenic porcine skin was found to survive much longer than the control after grafted to mouse wounds. We hope that this kind of transgenic porcine skin can be used in clinics in the near future.

#### Prevention and treatment of burn infections

##### Composing the clinical guidelines to prevent and treat the burn infection

To standardize the clinical prevention and treatment for burn infections, experts in the IBR and colleagues from other centers proposed several guidelines based on our own experiences and evidence. For post-burn sepsis, we raised the diagnostic criteria and treatment guidelines [30, 31]. The diagnostic criteria mainly include the infection of burned wounds, the pathogen diagnosis, and the systemic response. Furthermore, the treatment guidelines include infectious source control, rational use of antibiotics, continuous blood purification, application of glucocorticoids, immunomodulation, symptomatic and supportive treatment, and prevention of hospital-acquired infection. For burn invasive fungal infections, experts from the IBR and other 18 burn centers all over China composed the “Guideline for diagnosis, prophylaxis and treatment of invasive fungal infection post burn injury in China 2013” [32]. Host susceptibility, clinical manifestation, standardization of diagnosis, prevention, and treatment of fungal infection including surgical intervention are embodied.

##### Enterogenic infection

Generalized infection is one of the most serious complications during the treatment of burn patients. Large open wounds serve as classic portals of entry for a wide variety of infective microorganisms. However, studies conducted at the IBR were the first to confirm that a destroyed intestine was another important source of infection after burns. In 1962, we found that bacteria were detected in the patient blood before the corresponding bacteria emerged in a burn wound and the type of bacteria concentrated on the common bacteria in the intestine [33]. In the 1980s, animal experiments at our center showed that FITC-labelled *Pseudomonas aeruginosa*, *Bacteroides fragills*, and *Candida albicans* could be observed in the liver, spleen, lung, kidney, blood, and mesenteric lymph nodes in 3 h after animals were burned and received labelled organisms by gastric intubation [34, 35]. The pathogens could directly pass through the injured gut lines or were indirectly enwrapped by the intestinal epithelial cells. Furthermore, the gut endotoxin could invade into the blood through the abnormal tight junctions between intestine epithelial cells. Shock could accelerate the intestinal organism invasion, and therefore, the successful resuscitation from burn shock could partially prevent infection. As a common intestinal complication after burns, the intestinal tract obstruction could also disturb the bacterial balance and promote bacteria invasion. Malnutrition, especially a lack of glutamine, could result in the shrinking of intestinal mucosa, which could lead to the translocation of bacteria under the stimulation of an endotoxin [36].

Our results support the concept that indigenous gastrointestinal flora or exogenous organisms colonizing the gut intestine tract may be a potential reservoir for systemic bacterial infection following thermal injury, endotoxemia, endotoxemia plus malnutrition, and intestinal tract obstruction. Therefore, we recommend that bacteria translocation from the gut should be prevented by the following methods: adequate fluid resuscitation, easing stress-induced damage, early enteral feeding, prevention of flora imbalance, and others.

#### Nutrition therapy for severe burns

##### Estimation of calorie requirement: the TMMU energy formula

The hypermetabolic status after burns determines the high calorie and energy requirements. The calorie is the main type of energy expenditure. Therefore, it is crucial to correctly and simply estimate the calorie requirement in burn patients. Indirect calorimetry and formula estimation are the two common methods of estimating calorie requirement [12]. As the most widely used method, the Curreri formula is based on the body weight and TBSA (Adult calorie requirement [kJ/d] = 104.6 kJ × Body weight [kg] + 167.4 kJ × TBSA%) [37]. However, energy expenditure is positively associated with the body surface area, not the body weight. Furthermore, the Curreri formula estimates much higher calorie requirements in patients with large TBSA and is not suitable for the Chinese population who is of a different race and has different dietary habits from people in western countries. Therefore, we developed the first calorie estimation formula in China according to the kinetics of energy expenditure in 105 burn patients and healthy volunteers [38], as Adult calorie requirement [kJ/d] = 4184 kJ × Body area (m^2^) + 104.6 kJ × TBSA%. To simplify the calculation of body area, we derive the formula for body area. Body area (m^2^) = [Body height (m) − 0.6] × 1.5 [39].

**Fig. 9:**
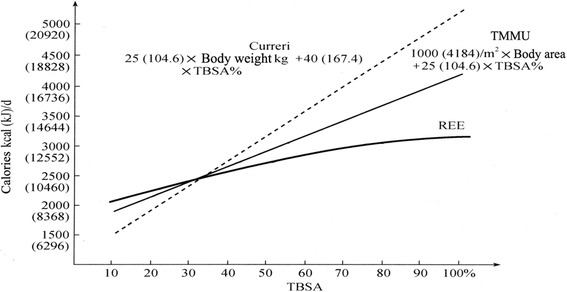
The comparison of the TMMU energy formula and Curreri formula with the indirect calorimetry. Adapted from references [12, 38]

To determine which formula is better, we compared the Curreri formula and the TMMU formula with the indirect calorimetry method [40]. The result is shown in Fig. 9. We found that the mean value of the TMMU formula was 20.5% higher than indirect calorimetry, and the mean value of the Curreri formula is 38.4% higher than indirect calorimetry. Furthermore, the migrated position of the TMMU formula is closer to indirect calorimetry than the Curreri formula. However, any calorie formula could only provide the “estimation” value, and adjustment is necessary based on the basic condition, wound healing, the current stage, and other factors.

##### Enterogenous hypermetabolism

Burns could provoke a significant stress response, and hypermetabolism is a hallmark of the stress response. Burned wounds and injured organs are the two main sources of hypermetabolism. Based on the series of research, we are the first to provide evidence that the intestinal tract, as the biggest endocrine and immune organ, is the main source of burn hypermetabolism and propose the concept of “enterogenous hypermetabolism” [41]. Ischemia, the disorder of energy metabolism, and the inhibition of repair and proliferation in the intestinal tract together lead to the destroyed structure and function of the intestinal tract and the organism or endotoxin translocation. Specifically, the neuroendocrine function and immune function were simultaneously hurt. These pathological changes resulted in the activation of the Kupffer cells and the secretion of inflammatory factors, hormones, transmits, and cytokines, which contributed to the increased catabolism of muscle and the hypermetabolism status of the whole body [42].

##### Early enteral nutrition support

To find a better method of nutrition support, we studied the influence of different nutritional support on enterogenous hypermetabolism [43, 44]. Our results showed that compared with the delayed enteral feeding (starting after 72 h) and early parenteral feeding, the early enteral feeding could significantly protect the function and structure of the intestinal tract and alleviate the hypermetabolism. Furthermore, glutamine [45, 46], intestinal trefoil factor [47–51], arginine acetate [52–54], and glucagon-like peptide 2 [55] could also exert a protective role in burn hypermetabolism. We also developed the commercial glutamine granules (Ankaishu), which have been widely used in the treatment of burn patients [56, 57].

#### Organ support therapies

Organ dysfunction is the second leading cause of deaths in severe burn patients. The most common organs that were impaired in burn patients were the lungs (56.1%), heart (26.0%), and kidney (20.2%). Therefore, organ support therapies are important in the treatment of burns.

##### Respiratory support

**Fig. 10:**
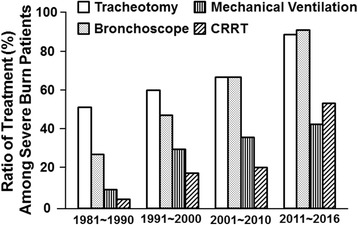
The respiratory and kidney supports among major burn patients (≥70% TBSA)

Respiratory failure is common in severe burns, especially in patients with inhalation injury. The respiratory support mainly included fiber-optic bronchoscopy, tracheotomy, and mechanical ventilation. The rates of tracheotomy and mechanical ventilation in severe burn patients (≥70% TBSA) were 52.0 and 28.0%, respectively, in the 1980s and dramatically increased to 90.3 and 92.3%, respectively, in the 2010s. Fiber-optic bronchoscopy can be used for the diagnosis and airway lavage treatment of inhalation injury [58]. The application of fiber-optic bronchoscopy in our institute started in the 1980s, and the utilization ratio in severe burn patients has risen gradually over the past three decades (Fig. 10).

##### Continuous renal replacement therapy (CRRT)

Increased cytokines are not only the bridge between burn infection and MODS but also the amplifier of burn inflammatory responses. CRRT was used in burn patients as support treatment for acute renal failure in the 1990s [59]. Our study showed that CRRT could remove plasma endotoxins and inflammatory cytokines (i.e., TNF-alpha, IL-1beta, IL-6, and IL-8) and exerted the role of “blood purification” [60, 61]. Meanwhile, early CRRT had no obvious effect on the vital signs, fluid input volumes, urine output, and platelet numbers [62, 63]. Furthermore, CRRT could improve the lung function in the burn patients with acute respiratory distress syndrome [64] and decrease the rate of sepsis, MODS, and mortality [62]. Therefore, CRRT is a safe and effective approach to prevent and treat burn infection and sepsis, especially in severe burn patients. In fact, the percentage of patients undergoing CRRT in severe burn patients (>70% TBSA) noticeably increased from 4.0% in the 1980s to approximately 18.0% in 1990s, 19.8% in the 2000s, and 55.3% in the 2010s (Fig. 10).

#### Rehabilitation

Frankly, the Chinese burn centers did not take rehabilitation seriously before the 1990s. However, our institute is the pioneer of implementing early rehabilitation for burn patients in Mainland China. Since 1995, we have had rehabilitation specialists for our burn patients, and since 2011, we have had a special burn rehabilitation center and team composed of 2 doctors, 12 rehabilitation therapists, and 1 psychological counselor. From then on, the methods of rehabilitation have gradually become diversified and professional. The provided rehabilitation includes not only anti-contracture positioning but also passive training (i.e., positioning, splinting, and passive range of motility (ROM) exercises) and mobility training (i.e., active ROM exercises, transfer training, and tilt table training) [65]. To raise the doctors’ awareness of rehabilitation and promote the development of rehabilitation in China, experts in our institute, who are the key members of the Chinese Burn Association and the Chinese Association of Burn Surgeons, organized the guidelines for Chinese burn rehabilitation in 2015 [66]. With the help of this guideline and the large number of burned patients, the burn rehabilitation center would eventually develop rapidly in our institute.

## Conclusions

IBR is one of the earliest burn centers set up in China. Since its establishment, the founder, Professor Li Ao, led his colleagues to be devoted to exploring the treatment and research of burns. During the past roughly 60 years, many achievements have been acquired, such as the Chinese Rule of Nine, Chinese fluid resuscitation formula for major burns, and Chinese protocol of nutrition support. Moreover, we also composed the protocols of infection prevention and treatment, rehabilitation, and pain control for burns, among other protocols during the past decades. All of the protocols have been widely applied in main China up to now, which contributes to the conspicuous treatment effect of burns in China. The progresses enrich and compose the Chinese modern burn medicine. Professor Li Ao and his colleagues received many honors and academic awards for their great accomplishment. However, there are still lots of problems such as lack of a national burn registry system, poor effect of scar prevention and treatment, and low efficiency of burn prevention in society, which are waiting for us and our colleagues to resolve.

## Abbreviations

CRRT: Continuous renal replacement therapy
IBR: Institute of Burn Research
MODS: Multiple organ dysfunction syndrome
MOF: Multiple organ failure
ROM: Range of motility
SIRS: Systemic inflammatory response syndrome
TBSA: Total body surface area
TMMU: Third Military Medical University
